# Parametric Analysis and Experimental Verification of a Hybrid Vibration Energy Harvester Combining Piezoelectric and Electromagnetic Mechanisms

**DOI:** 10.3390/mi8060189

**Published:** 2017-06-18

**Authors:** Zhenlong Xu, Xiaobiao Shan, Hong Yang, Wen Wang, Tao Xie

**Affiliations:** 1 School of Mechanical Engineering, Hangzhou Dianzi University, Hangzhou 310018, China; xzl@hdu.edu.cn (Z.X.); wangwn@hdu.edu.cn (W.W.); 2 School of Mechatronics Engineering, Harbin Institute of Technology, Harbin 150001, China; shanxiaobiao@hit.edu.cn; 3 College of Environmental and Resource Sciences, Zhejiang University, Hangzhou 310029, China; hyang_zju@163.com

**Keywords:** hybrid energy harvester, piezoelectric, electromagnetic, approximate distributed-parameter model, parametric analysis

## Abstract

Considering coil inductance and the spatial distribution of the magnetic field, this paper developed an approximate distributed-parameter model of a hybrid energy harvester (HEH). The analytical solutions were compared with numerical solutions. The effects of load resistances, electromechanical coupling factors, mechanical damping ratio, coil parameters and size scale on performance were investigated. A meso-scale HEH prototype was fabricated, tested and compared with a stand-alone piezoelectric energy harvester (PEH) and a stand-alone electromagnetic energy harvester (EMEH). The peak output power is 2.93% and 142.18% higher than that of the stand-alone PEH and EMEH, respectively. Moreover, its bandwidth is 108%- and 122.7%-times that of the stand-alone PEH and EMEH, respectively. The experimental results agreed well with the theoretical values. It is indicated that the linearized electromagnetic coupling coefficient is more suitable for low-level excitation acceleration. Hybrid energy harvesting contributes to widening the frequency bandwidth and improving energy conversion efficiency. However, only when the piezoelectric coupling effect is weak or medium can the HEH generate more power than the single-mechanism energy harvester. Hybrid energy harvesting can improve output power even at the microelectromechanical systems (MEMS) scale. This study presents a more effective model for the performance evaluation and structure optimization of the HEH.

## 1. Introduction

Vibration energy harvesting technology, which converts the ambient vibration energy into electric energy, has drawn much attention in recent years. It is considered as a promising solution to power the low-power portable microelectronic devices and wireless sensor networks. The conventional conversion mechanisms comprise piezoelectric [1], electromagnetic [2], electrostatic [3] and magnetostrictive [4] types. It is a challenge for researchers to design the vibration energy harvester (VEH) with a broad operating frequency bandwidth and outstanding energy density. Because many reported VEHs are based on the principle of a linear single-degree-of-freedom (SDOF) system, only when the resonant frequencies match the excitation frequencies can they achieve the optimum generating performance. However, the ambient vibration frequency is broadband and random. To improve the performance of the VEH, hybrid energy harvesting technology combining piezoelectric and electromagnetic mechanisms is proposed. It shows an increasing trend and attracts more and more attention from scholars.

Generally, the hybrid energy harvester (HEH) is transformed from the piezoelectric energy harvester (PEH) by replacing the proof mass with a permanent magnet and adding an induction coil. The PEH generates electricity by means of the piezoelectric effect. The magnet is used to tune the resonant frequency and amplify the deformation of the piezoelectric element. Meanwhile, the relative motion between magnet and coil can induce electric current in the coil due to Faraday’s law of electromagnetic induction. Both the magnet and induction coil are the components of the electromagnetic energy harvester (EMEH). Considering different vibration sources and application backgrounds, scholars conducted a series of structural designs for the HEH, such as wearable HEH [5], cantilever-type HEH [6,7], multimodal HEH [8,9,10], multi-frequency HEH [11], nonlinear HEH [12,13] and frequency up-converted HEH [14,15]. In a word, the beam structure is the most typical configuration of the reported HEH. With the development of microelectromechanical systems (MEMS), the dimension of the HEH shrinks from meso to micro size [16]. In order to evaluate the generating performance of micro-scale HEH, it is meaningful to investigate the scaling effects on the mechanical and electrical properties, which has not been reported up to now.

In addition, some scholars also make efforts to develop an effective theoretical model of the HEH to predict the generating performance and analyze the effects of parameters on the energy harvesting characteristics. Mostly, the HEH is simplified to be a spring-mass-damper system, and the lumped-parameter theoretical model [17] is established. However, many models [5,6,7,9,10] ignored the effects of piezoelectric and electromagnetic coupling coefficients on the effective stiffness of the system. Both Li et al. [18] and Shan et al. [19] ignored the influence of coil inductance. The approximate distributed-parameter model [12,20] derived from the energy method is another common model, which is more accurate than the lumped-parameter one. It takes into account the effects of mode shape, strain distribution, distributed mass and stiffness on the performance of the energy harvester. In the process of mathematical modeling, the electromagnetic coupling coefficient is mostly oversimplified and considered to be a constant. The effects of the nonlinear and linear electromagnetic coupling coefficients on the output power are not clear. Moreover, the effect of the coil inductance mostly is not taken into account. Therefore, it is disadvantageous for the performance evaluation and structure optimization of the HEH based on those theoretical models.

In this paper, a hybrid piezoelectric-electromagnet energy harvester is modeled, theoretically analyzed and experimentally tested. The objective of this paper is to develop an approximate distributed-parameter theoretical model of the HEH by considering the coil inductance and spatial distribution of magnetic field and to analyze comprehensively the effects of key parameters on the generating performance.

## 2. Modeling

**Figure 1:**
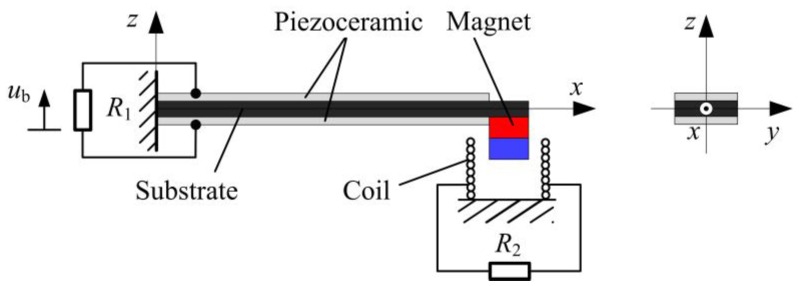
Schematic diagram of the hybrid energy harvester.

Based on the previous research articles, a typical HEH with the cantilever-beam structure is designed, as depicted in Figure 1. It comprises a bimorph piezoelectric cantilever beam with a permanent magnet as the tip mass and a cylindrical induction coil attached on the frame. The piezoelectric beam is fixed on the base, whose oscillation is harmonic. The piezoceramic operates in the *d*_31_ mode. Both piezoceramic layers are assumed to be perfectly bonded to both sides of the substrate, respectively. They are oppositely poled in the thickness direction and connected in series. The conductive electrodes fully cover the top and bottom surfaces of the piezoceramic layers. The proof magnet is considered as a mass point without the rotary inertia. Under the small-amplitude oscillation, the Euler–Bernoulli beam theory is applicable to the piezoelectric beam. As a consequence, the rotary inertia and shear deformation of the beam are neglected. In addition, the coil and magnet are axially aligned.

The electromechanical coupling model of the HEH can be derived by the energy method [21,22]. According to Hamilton’s principle, the energies of the electromechanical system satisfy the following equation:

(1) $$\int_{t_{1}}^{t_{2}}\left[\delta \left(T-U\right)+\delta W_{\mathrm{nc}}\right]dt=0$$

where *T*, *U* and *W*_nc_ denote the kinetic energy, potential energy and virtual work of the system, respectively. *δ* is the variational operator.

When the HEH is subjected the transverse harmonic excitation along the *z*-axis, only the transverse displacement is considered. The kinetic energy *T* of the HEH is the sum of the translational kinetic energies of the cantilever beam and magnet, which is defined as:

(2) $$T=\frac{1}{2}\int_{V_{s}}\rho_{s}{\dot{u}}^{t}\dot{u}dV_{s}+\frac{1}{2}\int_{V_{p}}\rho_{p}{\dot{u}}^{t}\dot{u}dV_{p}+\frac{1}{2}M_{t}\dot{u}{\left(L\right)}^{t}\dot{u}(L)$$

where *V* and *ρ* represent the volume and density, respectively. The subscripts “s” and “p” denote the substrate and piezoceramic, respectively. *M*_t_ is the mass of the magnet. *L* is the length of the beam. *u*(*x*, *t*) is the displacement of the piezoelectric beam relative to the base at the axial position *x* and time *t*. Dots indicate the time derivatives. Therefore, $\dot{u}$ is the velocity. The superscript “*t*” denotes the transpose of the matrix.

The potential energy *U* consists of four terms: the elastic potential energy stored in the substrate and piezoceramic layers, electric energy stored in the piezoceramic layers and the magnetic co-energy stored in the induction coil. It is given by:

(3) $$U=\frac{1}{2}\int_{V_{s}}S^{t}TdV_{s}+\frac{1}{2}\int_{V_{p}}S^{t}TdV_{p}+\frac{1}{2}\int_{V_{p}}E^{t}DdV_{p}+\frac{1}{2}L_{c}{\dot{Q}}^{2}$$

where *S*, *T*, *E* and *D* are the strain, stress, electric field and electric displacement, respectively. *Q* is the charge passing through the coil. *L*_c_ is the inductance of the coil.

The virtual work *W*_nc_ applied to the HEH contains the mechanical work done by electromagnetic force, base excitation force, mechanical damping force and electrical work due to the charges. Hence, the virtual work can be expressed as:

(4) $$\begin{array}{ll} \delta W_{\mathrm{nc}}= & -\delta u{\left(L\right)}^{t}M_{t}{\ddot{u}}_{b}-\int_{V_{s}}\delta u^{t}\rho_{s}{\ddot{u}}_{b}dV_{s}-\int_{V_{p}}\delta u^{t}\rho_{p}{\ddot{u}}_{b}dV_{p}-\int_{0}^{L}C\dot{u}\delta udx\\ & -\delta Q^{t}(R_{c}+R_{2})\dot{Q}+\sum_{j=1}^{nq}\delta \varphi_{j}q_{j}+\theta_{e}\dot{Q}\delta u(L) \end{array}$$

where *u*_b_(*t*) is the base displacement and ${\ddot{u}}_{b}$ is the acceleration. *C* denotes the viscous damping, which can be experimentally determined by the logarithmic decrement method [23]. *R*_c_ and *R*_2_ are the internal resistance of the coil and the external load resistance connected to the coil, respectively. $\varphi_{j}=\varphi (x_{j},t)$ is the scalar electrical potential for each of the *nq* electrode pairs at position *x_j_*. *q_j_* is the charge extracted from the corresponding electrode pairs. *θ*_e_ is the electromagnetic coupling coefficient.

Due to the low frequency of the ambient excitation, the first mode is our research focus. Based on the small-signal constitutive equations of piezoelectricity [24], the relationship between the stress and strain of the substrate, the Rayleigh–Ritz approach and the Euler–Bernoulli beam assumption, the modal electromechanical coupling equations of the HEH are given by:

(5) $$\left\{ \begin{array}{l} M\ddot{r}+C\dot{r}+Kr-\theta_{p}v-\theta_{\mathrm{em}}I_{2}=-B_{f}{\ddot{u}}_{b}\\ \theta_{p}\dot{r}+C_{p}\dot{v}+v/R_{1}=0\\ \theta_{\mathrm{em}}\dot{r}+L_{c}{\dot{I}}_{2}+(R_{c}+R_{2})I_{2}=0 \end{array}\right.$$

where $v=R_{1}\dot{q}$ is the voltage across the piezoelectric beam. *R*_1_ is the external load resistance connected to the beam. *I*_2_ is the current in the coil. Note that *u*(*x*, *t*) = *ψ*_r_(*x*)*r*(*t*), where *ψ*_r_(*x*) and *r*(*t*) denote the mode shape and mechanical temporal coordinate, respectively. *M*, *K*, *θ*_p_, *θ*_em_, *B*_f_ and *C*_p_ are the effective mass, effective stiffness, piezoelectric coupling term, electromagnetic coupling term, forcing coefficient and capacitance, respectively. *θ*_em_ is equal to the product of *θ*_e_ and *ψ*_r_(*L*).

The calculation of the electromagnetic coupling coefficient *θ*_e_ is related to the accuracy of the mathematical model and the optimization of system parameters. In this paper, it is established based on the magnetic dipoles model, which has been reported in our previous study [11]. The result is expressed as:

(6) $$\begin{array}{ll} \theta_{e}= & -\frac{B_{r}V_{m}f_{c}N}{2A_{c}}[\ln\frac{R_{i}+\sqrt{R_{i}^{2}+{\left(z_{2}-h_{c}\right)}^{2}}}{R_{o}+\sqrt{R_{o}^{2}+{\left(z_{2}-h_{c}\right)}^{2}}}+\ln\frac{R_{o}+\sqrt{R_{o}^{2}+z_{2}^{2}}}{R_{i}+\sqrt{R_{i}^{2}+z_{2}^{2}}}\\ & +\frac{R_{o}}{\sqrt{R_{o}^{2}+{\left(z_{2}-h_{c}\right)}^{2}}}-\frac{R_{o}}{\sqrt{R_{o}^{2}+z_{2}^{2}}}-\frac{R_{i}}{\sqrt{R_{i}^{2}+{\left(z_{2}-h_{c}\right)}^{2}}}+\frac{R_{i}}{\sqrt{R_{i}^{2}+z_{2}^{2}}}] \end{array}$$

where *B_r_* and *V*_m_ are the residual magnetic flux density and volume of the magnet, respectively. *R*_i_, *R*_o_, *h*_c_, *f*_c_ and *N* are the inner radius, outer radius, height, fill factor and turns of the induction coil, respectively. *A*_c_ = (*R*_o_ − *R*_i_)*h*_c_ is the cross-section area of the coil. *z*_2_ is the position coordinate of the magnet core relative to the coil. It can be seen that there is a nonlinear change of *θ*_e_ with the change of magnet position *z*_2_. This model takes the spatial distribution of the magnetic field into account.

When the HEH is excited by the harmonic vibration, the beam-tip displacement relative to the base is *u*(*L*). Therefore, *z*_2_ can be expressed as *z*_2_ = *u*(*L*) + *z*_0_, where *z*_0_ is the position coordinate of the magnet core at static balance. A Taylor series expansion of *θ*_e_ about *z*_0_ is:

(7) $$\theta_{e}=\sum_{i=0}^{\infty }{\frac{\partial^{i}\theta_{e}}{\partial z_{2}^{i}}|}_{z_{2}=z_{0}}\cdot \frac{u^{i}(L)}{i!}$$

For convenience, *θ*_e_ can be simplified to *θ*_e_(*z*_0_) in the linearized model under small signal excitation.

Defining a state vector $X={\left[\begin{array}{cccc} X_{1} & X_{2} & X_{3} & X_{4} \end{array}\right]}^{t}={\left[\begin{array}{cccc} r & \dot{r} & v & I_{2} \end{array}\right]}^{t}$ (*t* denotes the transpose of the vector here), Equation (5) can be written in the state space form as:

(8) $$\dot{X}=\left[\begin{array}{c} X_{2}\\ -\frac{K}{M}X_{1}-\frac{C}{M}X_{2}+\frac{\theta_{p}}{M}X_{3}+\frac{\theta_{\mathrm{em}}}{M}X_{4}-\frac{B_{f}}{M}{\ddot{u}}_{b}\\ -\frac{\theta_{p}}{C_{p}}X_{2}-\frac{1}{C_{p}R_{1}}X_{3}\\ -\frac{\theta_{\mathrm{em}}}{L_{c}}X_{2}-\frac{R_{c}+R_{2}}{L_{c}}X_{4} \end{array}\right]$$

The output average power delivered to the external loads *R*_1_ and *R*_2_ is respectively given as:

(9) Pnp=1T∫0Tv2R1dt

(10) $$P_{\mathrm{ne}}=\frac{1}{T}\int_{0}^{T}I_{2}^{2}R_{2}dt$$

where *T* = 2*π*/*ω* is the cycle of the base excitation. *ω* is the angular velocity.

The total output power of the HEH with nonlinear *θ*_e_ is:

(11) $$P_{1}=P_{\mathrm{np}}+P_{\mathrm{ne}}$$

When using the linearized model of *θ*_e_, the analytical solutions of the amplitude for relative displacement, voltage across the load resistance *R*_1_ and current through the coil can be derived from Equation (5), as shown below:

(12) $$\left|r\right|=\frac{\left|{\ddot{u}}_{b}\right|B_{f}}{K}\cdot {\left[{\left(1-\Omega^{2}+\frac{\kappa_{p}^{2}\lambda_{p}^{2}\Omega^{2}}{1+\lambda_{p}^{2}\Omega^{2}}+\frac{\kappa_{e}^{2}\lambda_{e}^{2}\Omega^{2}}{1+\lambda_{e}^{2}\Omega^{2}}\right)}^{2}+{\left(2\zeta_{m}\Omega +\frac{\kappa_{p}^{2}\lambda_{p}\Omega }{1+\lambda_{p}^{2}\Omega^{2}}+\frac{\kappa_{e}^{2}\lambda_{e}\Omega }{1+\lambda_{e}^{2}\Omega^{2}}\right)}^{2}\right]}^{-1/2}$$

(13) $$\left|v\right|=\frac{\theta_{p}}{C_{p}}\cdot \frac{\lambda_{p}\Omega }{\sqrt{1+\lambda_{p}^{2}\Omega^{2}}}\cdot \left|r\right|$$

(14) $$\left|I_{2}\right|=\frac{\theta_{\mathrm{em}}}{L_{c}}\cdot \frac{\lambda_{e}\Omega }{\sqrt{1+\lambda_{e}^{2}\Omega^{2}}}\cdot \left|r\right|$$

where Ω = *ω*/*ω*_1_ is the excitation frequency ratio. $\omega_{1}=\sqrt{K/M}$ is the first undamped natural frequency of the piezoelectric beam. $\kappa_{p}^{2}=\theta_{p}^{2}/\left(KC_{p}\right)$ and $\kappa_{e}^{2}=\theta_{\mathrm{em}}^{2}/\left(KL_{c}\right)$ are the effective electromechanical coupling coefficients for the PEH and EMEH, respectively. *λ*_p_ = *R*_1_*C*_p_*ω*_1_ and *λ*_e_ = *L*_c_*ω*_1_/(*R*_c_ + *R*_2_) are the normalized load resistances connected to the PEH and EMEH, respectively. $\zeta_{m}=C/\left(2\sqrt{KM}\right)$ is the mechanical damping ratio.

The instantaneous output power of the PEH is calculated as *P*_p_ = *v*^2^/*R*_1_, and that of EMEH is $P_{e}=I_{2}^{2}R_{2}$. Therefore, the total average output power of the HEH with linear *θ*_e_ is:

(15) $$P_{2}={\bar{P}}_{p}+{\bar{P}}_{e}=\frac{r_{\max}^{2}}{2}\left[\frac{K\omega_{1}\kappa_{p}^{2}\lambda_{p}\Omega^{2}}{1+\lambda_{p}^{2}\Omega^{2}}+\frac{K\omega_{1}\kappa_{e}^{2}\lambda_{e}\Omega^{2}}{1+\lambda_{e}^{2}\Omega^{2}}\cdot \left(1-\frac{\lambda_{e}}{\lambda_{c}}\right)\right]$$

where *λ*_c_ = *L*_c_*ω*_1_/*R*_c_ is the normalized internal resistance of the coil. *r*_max_ denotes the magnitude of the relative displacement.

The harvested vibration energy from the base excitation is ultimately dissipated due to the mechanical damping and electric resistances. We have to note that there is power loss due to the internal resistance of the coil. In general, this part is neglected by researchers [25,26]. The average power dissipated by the mechanical damping and internal resistance of the coil is respectively expressed as:

(16) $${\bar{P}}_{d}=\frac{\omega }{2\pi }\int_{0}^{2\pi /\omega }C{\dot{r}}^{2}dt=\frac{1}{2}C\omega^{2}r_{\max}^{2}$$

(17) $${\bar{P}}_{c}={\bar{P}}_{e}\cdot \frac{R_{c}}{R_{2}}$$

As a result, the energy conversion efficiency is obtained by:

(18) $$\eta =\frac{P_{2}}{P_{2}+{\bar{P}}_{d}+{\bar{P}}_{c}}$$

From Equation (12), it can be seen that the piezoelectric and electromagnetic coupling affect the effective stiffness and damping of the system. The effective damping of the HEH is:

(19) $$C_{\mathrm{eff}}=C+\frac{K\kappa_{p}^{2}\lambda_{p}}{\omega_{1}(1+\lambda_{p}^{2}\Omega^{2})}+\frac{K\kappa_{e}^{2}\lambda_{e}}{\omega_{1}(1+\lambda_{e}^{2}\Omega^{2})}=C+D_{p}+D_{\mathrm{em}}$$

where *D*_p_ and *D*_em_ are respectively defined as the piezoelectric and electromagnetic damping.

The power angular bandwidth [27] of the HEH can be derived by:

(20) $$\Delta \omega =2\zeta \omega_{1}=2(\zeta_{m}+\zeta_{e})\omega_{1}=\frac{C+D_{p}+D_{\mathrm{em}}}{M}$$

where *ζ* is the total damping ratio of the HEH. $\zeta_{e}=\left(D_{p}+D_{\mathrm{em}}\right)/\left(2\sqrt{KM}\right)$ denotes the electrical damping ratio, which is the sum of the piezoelectric and electromagnetic damping ratios.

## 3. Fabrication and Parametric Analysis

In this section, numerical and analytical solutions of the output power of the HEH for different excitation frequency ratios and accelerations are obtained by using MATLAB software (R2012b, MathWorks Inc., Natick, MA, USA) and compared firstly, so as to analyze the effect of electromagnetic coupling coefficient. The analytical solutions are used to investigate the influences of electric load resistances, electromechanical coupling factors, mechanical damping ratio, coil parameters and size scale on the generating characteristics and dynamic responses of the HEH with the acceleration of the harmonic base excitation 2 m/s^2^.

**Figure 2:**
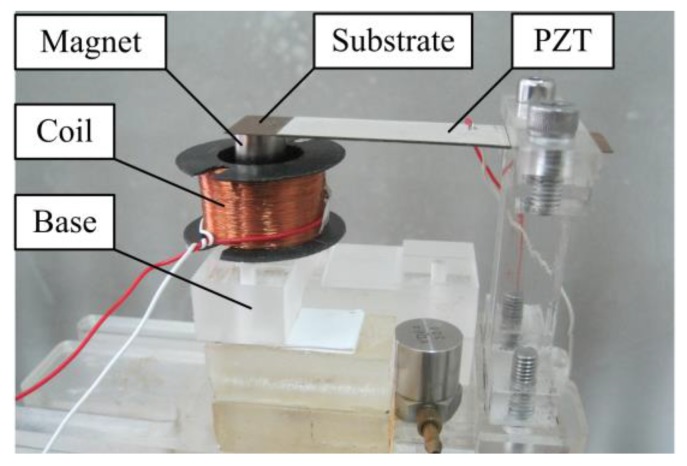
Prototype of the hybrid energy harvester (HEH).

Firstly, a meso-scale HEH prototype based on the proposed structure was fabricated, as shown in Figure 2. The bimorph piezoelectric cantilever beam is made of phosphor bronze substrate and two lead zirconate titanate (PZT-5H) layers. The magnet material is NdFeB (N35). The induction coil is wound with copper wire. The geometric and material parameters of the HEH are listed in Table 1, where $c_{11}^{E}$, *e*_31_ and $\varepsilon_{33}^{S}$ are experimentally obtained by using the impedance analyzer (Agilent 4294A). For the convenience of qualitative analysis, the magnetic core is located at the same height as the upper surface of the coil at the static state. The default of the mechanical damping ratio *ζ*_m_ is set to 0.02.

### 3.1. Comparison of Numerical and Analytical Solutions

**Figure 3:**
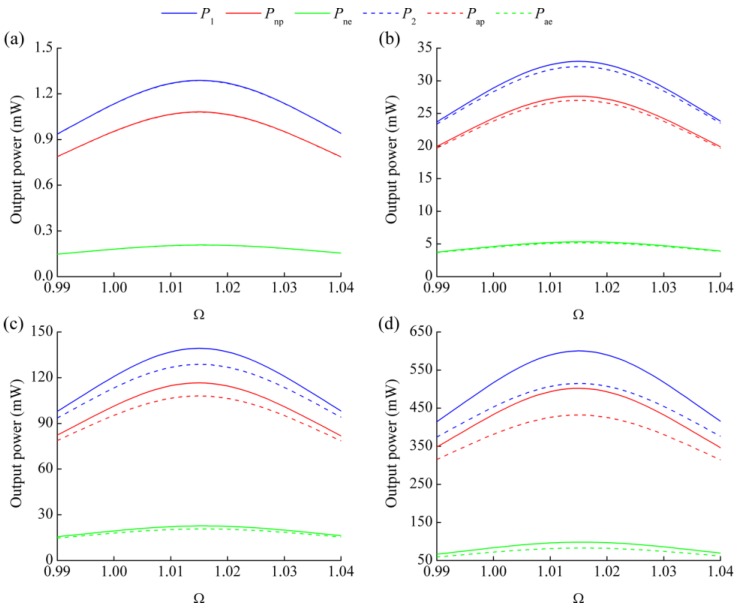
Output power versus excitation frequency ratio for different excitation accelerations: (**a**) 2 m/s^2^; (**b**) 10 m/s^2^; (**c**) 20 m/s^2^; (**d**) 40 m/s^2^.

Figure 3 shows the comparison of output power obtained from numerical and analytical solutions at 2-, 10-, 20- and 40-m/s^2^ excitation acceleration. Note that *P*_ap_ and *P*_ae_ denote the analytical results generated from PEH and EMEH subsystems, respectively. The values of *R*_1_ and *R*_2_ are 1/(*C*_p_*ω*_1_) and *R*_c_, respectively. Define *ω*_r_ as the resonant frequency of the HEH with load resistances and Ω_r_ = *ω*_r_/*ω*_1_ as the normalized resonant frequency. From Figure 3, we can see that the resonant frequency ratio Ω_r_ of HEH is not affected by the excitation acceleration and remains unchanged. However, the difference between the output power of numerical and analytical results gradually increases with the increment of excitation acceleration. This is due to the effect of electromagnetic coupling coefficient *θ*_e_. In the linearized model, *θ*_e_ is considered to be a constant, which is almost equal to the maximum value of nonlinear *θ*_e_ in the steady state. Consequently, the electromagnetic damping force induced by the linearized *θ*_e_ is much larger than that in the numerical model. The vibration response of piezoelectric beam is suppressed more obviously, which leads to the decrease of the output power from the PEH subsystem. Furthermore, the effect of vibration suppression is enhanced with the increasing of the excitation acceleration, resulting in the enlargement of the difference between numerical and analytical solutions. Although the output power generated from the EMEH subsystem is related with *θ*_e_, the relative velocity between magnet and induction coil is another determining factor. The magnet is slowed down along with the vibration suppression. Therefore, the output power of the EMEH subsystem also decreases. It is indicated that the linearized model of *θ*_e_ is more suitable to predict the generating performance of the HEH in the low excitation acceleration level.

**Figure 4:**
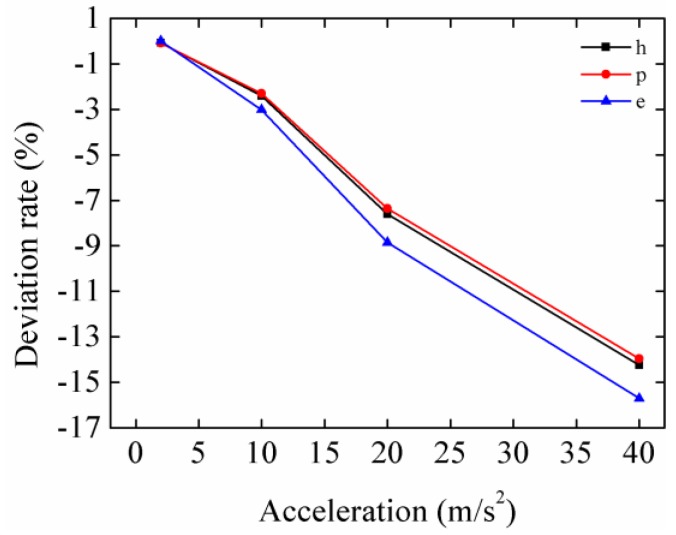
Deviation rates of the analytical values relative to numerical values. “h”, “p” and “e” in the legend represent the HEH, the piezoelectric energy harvester (PEH) subsystem and the electromagnetic energy harvester (EMEH) subsystem.

Table 2 lists the resonant frequency ratios and peak output power at different excitation accelerations obtained from numerical and analytical solutions. Figure 4 shows the deviation rates of analytical values relative to the numerical values. “h”, “p” and “e” in the legend represent the HEH, PEH subsystem and EMEH subsystem. The minus sign of the deviation rate indicates that the analytical value is less than the numerical one. Obviously, the deviation rates have a similar trend. Meanwhile, the peak power of the EMEH subsystem is the most affected. However, its magnitude is approximately one-fifth of that of the peak power generated from the piezoelectric subsystem. Therefore, linearized *θ*_e_ has almost the same effect on the peak power of the HEH and PEH subsystem.

### 3.2. Effects of Load Resistances on the Performance of the Hybrid Energy Harvester (HEH)

Based on the previous analysis, there is little difference between analytical and numerical solutions at low-level excitation acceleration. Consequently, the analytical solutions will be used in the later sections, and the excitation acceleration is set to 2 m/s^2^.

As defined in the modeling section, *λ*_p_ and *λ*_e_ are the normalized electric resistances connected to the piezoelectric layers and induction coil, respectively. From Equation (12), the equivalent stiffness of the system can be expressed as:

(21) $$K_{\mathrm{eff}}=K+\frac{\theta_{p}^{2}R_{1}^{2}C_{p}\omega^{2}}{1+R_{1}^{2}C_{p}^{2}\omega^{2}}+\frac{\theta_{\mathrm{em}}^{2}L_{c}\omega^{2}}{L_{c}^{2}\omega^{2}+{\left(R_{2}+R_{c}\right)}^{2}}$$

It can be seen that load resistances can change the equivalent stiffness, as well as the resonant frequency.

**Figure 5:**
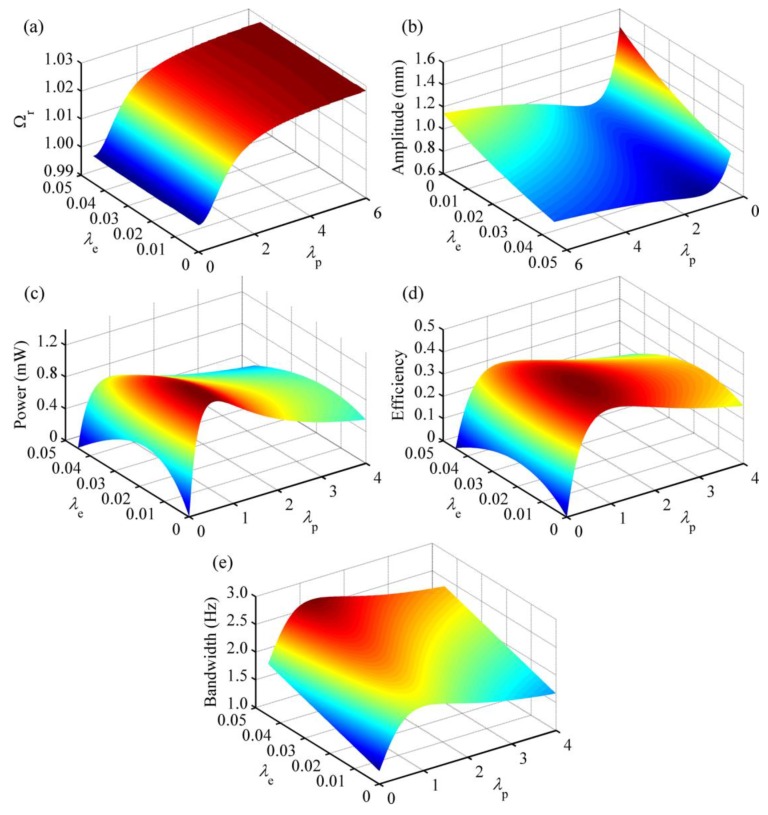
Performances of the HEH for different normalized load resistances *λ*_p_ and *λ*_e_: (**a**) normalized resonant frequency Ω_r_; (**b**) free-end displacement amplitude of the piezoelectric beam at the resonant frequency; (**c**) output power; (**d**) energy conversion efficiency; (**e**) operating frequency bandwidth.

Figure 5 shows the effects of normalized load resistances on the performance of the HEH. Obviously, *λ*_p_ plays a greater role in tuning the resonant frequency, as displayed in Figure 5a. With the increasing of *λ*_p_, the resonant frequency gradually rises and tends to a stable value. However, the influence of *λ*_e_ seems to be negligible. Therefore, during the impedance matching of the experiment, the load resistance of the PEH subsystem should be preferentially determined, then that of the EMEH part. When *λ*_p_ = 0 and *λ*_e_ = 0, the piezoelectric layers are short-circuit, and the coil is open-circuit. At this time, Ω_r_ = 0.9996, which denotes the normalized damped natural frequency and is named as the short-circuit resonant frequency sometimes [28]. When *λ*_p_ = 6, the piezoelectric layers are close to the open-circuit condition. The open-circuit resonant frequency is 1.0284 when the coil is open-circuit. This value is determined by the piezoelectric coupling term.

In Figure 5b, it can be seen that both load resistances affect the free-end displacement amplitude of the piezoelectric beam at the resonant frequency. With the increment of *λ*_p_, this value first declines and then bounces back. However, it steadily decreases with the rising of *λ*_e_. When *λ*_p_ = 1.02 and *λ*_e_ = *λ*_c_, the amplitude reaches the lowest point 0.6189 mm, which is 42.86% the maximum value (1.4441 mm). It reveals that extracting electrical energy can suppress the vibration of the beam. Letting the excitation frequency ratio Ω = 1, Figure 5c illustrates the output power of the HEH for different load resistances. It is clear that the output power first rises up and then falls off with the increasing of one load resistance, when the other one is kept constant. It reaches the maximum value 1.3730 mW when *λ*_p_ = 0.55 and *λ*_e_ = 0.005. According to the figure, we can conclude that hybrid energy harvesting provides an increment of output power, based on the parameters defined before. The energy conversion efficiency has the similar tendency as that of output power, as shown in Figure 5d. It reaches the highest 45.19% when *λ*_p_ = 1 and *λ*_e_ = 0.012. Comparison of Figure 5c,d shows that the matched load resistances for the optimal output power and energy conversion efficiency are not consistent. This is because the output power is related to the product of amplitude and efficiency. Furthermore, the optimal output power and energy conversion efficiency do not imply an optimal vibration suppression effect.

Figure 5e represents the varying of the operating frequency bandwidth. The bandwidth rises up with the increase of *λ*_e_. However, it first increases and then declines when *λ*_p_ = 1. Due to the coupling of electromagnetic and piezoelectric energy harvesting, the electrical damping of the system increases, resulting in widening of the operating frequency bandwidth.

### 3.3. Effects of Electromechanical Coupling Factors on the Performance of the HEH

In previous literature [29,30], scholars took $\kappa_{p}^{2}/\zeta_{m}$ as the indicator of the piezoelectric coupling effect. In this paper, we define the piezoelectric coupling factor $\sigma_{p}=\kappa_{p}^{2}/\zeta_{m}$. Similarly, we take electromagnetic coupling factor $\sigma_{e}=\kappa_{e}^{2}/\zeta_{m}$ as the indicator of the electromagnetic coupling effect. Based on the parameters defined before, the values of *σ*_p_ and *σ*_e_ are about 3.0 and 25.0, respectively. Ω is still set to one.

**Figure 6:**
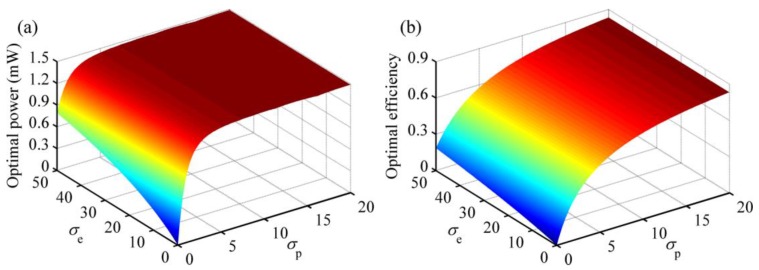
Optimal output power and energy conversion efficiency for different coupling factors *σ*_p_ and *σ*_e_: (**a**) output power; (**b**) energy conversion efficiency.

Figure 6 plots the optimal output power and energy conversion efficiency of the HEH with matched load resistances for different *σ*_p_ and *σ*_e_. The power climbs sharply at first and then slows down as *σ*_p_ increases. There is also a growing tendency of the optimal power with the increase of *σ*_e_, although the influence of *σ*_e_ almost fades off when *σ*_p_ is larger than eight. Apparently, *σ*_p_ exerts stronger influence on the power than *σ*_e_. In a word, when the piezoelectric coupling effect is weak or medium, HEH generates more power than the single-mechanism energy harvester. With the enhancement of coupling effects, the efficiency also steadily increases. According to the figure, we can conclude that hybrid energy harvesting mechanism contributes to improving the energy conversion efficiency of VEH.

### 3.4. Effect of Mechanical Damping Ratio on the Performance of the HEH

**Figure 7:**
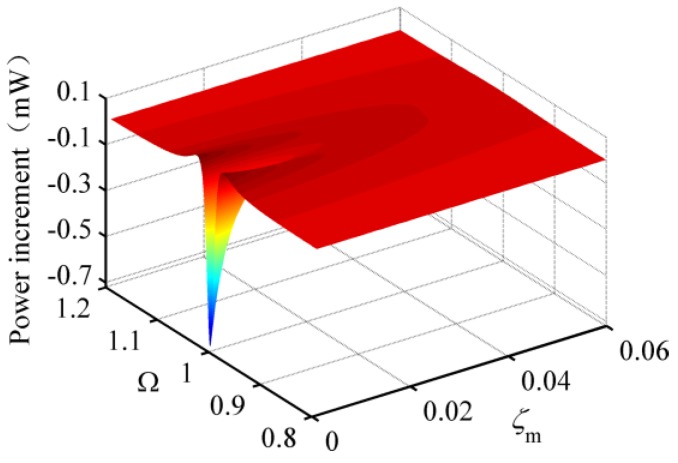
The output power increment of the HEH relative to the conventional PEH with matched load resistances for different Ω and *ζ*_m_.

Mechanical damping can consume the energy of the HEH and limits the generating performance. To observe the effect of mechanical damping, Figure 7 provides the output power increment of the HEH relative to the conventional PEH with matched load resistances for different Ω and *ζ*_m_. Both matched values of *λ*_p_ connected to HEH and PEH are 0.55. The matched *λ*_e_ is 0.005. It can be seen from the figure that when *ζ*_m_ < 0.041, there are two peaks in the power increment, corresponding to the vicinity of excitation frequency ratios of 0.98 and 1.03. On the contrary, there is only one peak around Ω = 1.03. The appearance of two peaks is due to the broader bandwidth of the HEH. In addition, the minimum power increment dramatically reduces to the negative value with the decrease of *ζ*_m_. It implies that the peak output power of the PEH is larger than that of the HEH under the condition of very small mechanical damping. When *ζ*_m_ is bigger than 0.015, the generating performance of the HEH is definitely better than that of the PEH. Overall, the mechanical damping ratio can affect the superiority of the HEH to the PEH.

### 3.5. Effects of Coil Parameters on the Performance of the HEH

**Figure 8:**
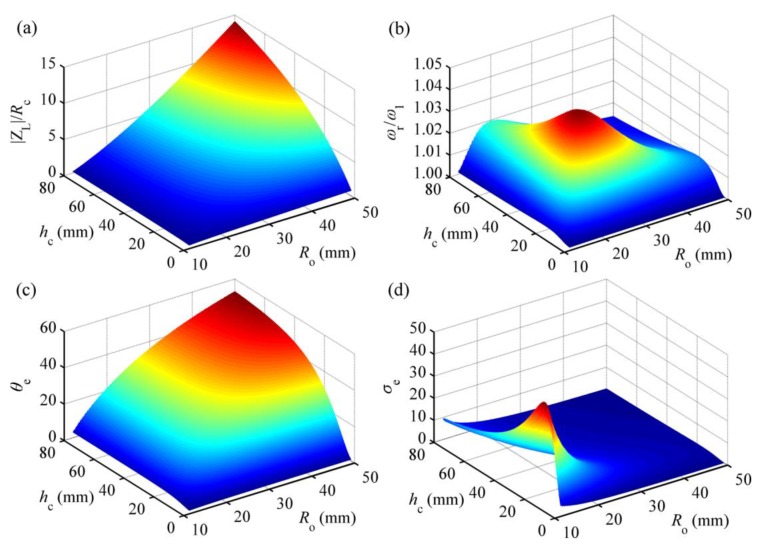
Performance of the HEH for different *h*_c_ and *R*_o_: (**a**) ratio of inductive reactance |Z_L_| to internal resistance *R*_c_ of the coil; (**b**) ratio of resonant frequency *ω*_r_ to undamped natural frequency *ω*_1_; (**c**) electromagnetic coupling coefficient *θ*_e_; (**d**) electromagnetic coupling factor *σ*_e_.

In order to evaluate the effects of the induction coil parameters on the generating performance of the HEH, we analyzed the effects of height *h*_c_ and outer radius *R*_o_ of the coil on the inductive reactance |Z_L_|, resonance frequency *ω*_r_, electromagnetic coupling coefficient *θ*_e_ and electromagnetic coupling factor *σ*_e_, as shown in Figure 8.

Note that |Z_L_| = *ωL*_c_. Considering the frequencies of most vibration sources in the environment below 200 Hz [31], the excitation frequency in the analytical model is assumed to be 200 Hz. For a certain geometry of the coil, the copper wire is supposed to be tightly wound and entirely fill the coil volume. The external load resistance *R*_2_ is equal to *R*_c_. When *h*_c_ and *R*_o_ increase, the coil turns and *R*_c_ will increase, as well as the ratio of |Z_L_| to *R*_c_. As designed in this paper, *R*_o_ is 13.15 mm, and *h*_c_ is 17 mm. The corresponding ratio is about 0.35, which is much higher than the actual value. If the matched load resistance *R*_2_ is taken into account, the ratio of |Z_L_| to (*R*_c_ + *R*_2_) will be close to 0.18. In an integral energy harvesting system, the proportion of |Z_L_| relative to the total impedance of the system will further drop due to the impedance introduced by the external circuit. In Figure 8b, the load resistance *R*_1_ is set zero to exclude the influence of the piezoelectric coupling term on the resonant frequency. As can be seen, the ratio reaches the maximum value 1.05 when *h*_c_ = 21.1 mm and *R*_o_ = 21.4 mm. Hence, the resonant frequency of the vibration energy harvester can be tuned by changing the parameters of the induction coil. However, the frequency tuning range is so limited that many scholars neglected the effect of inductive reactance on the resonant frequency for the simplification.

There is a notable distinction between electromagnetic coupling coefficient *θ*_e_ and coupling factor *σ*_e_ for different *h*_c_ and *R*_o_. *θ*_e_ gradually increases with the increase of *h*_c_ or *R*_o_. As reported before [11], the inductive electromotive force is proportional to *θ*_e_. However, this does not mean that the higher *θ*_e_ is, the better the generating performance of the HEH is. *σ*_e_ shows a downward trend with the increase of *R*_o_ and reaches the peak when *h*_c_ = 13.1 mm and *R*_o_ = 12.3 mm. This is mainly because the increase of *h*_c_ and *R*_o_ induces the increase of inductance of the coil. Referring to Figure 6, the higher the value of *σ*_e_ is, the more electric power that can be generated. Therefore, the coil parameters of the HEH should be optimized according to the variation of *σ*_e_.

### 3.6. Scaling Effects on the Performance of the HEH

**Figure 9:**
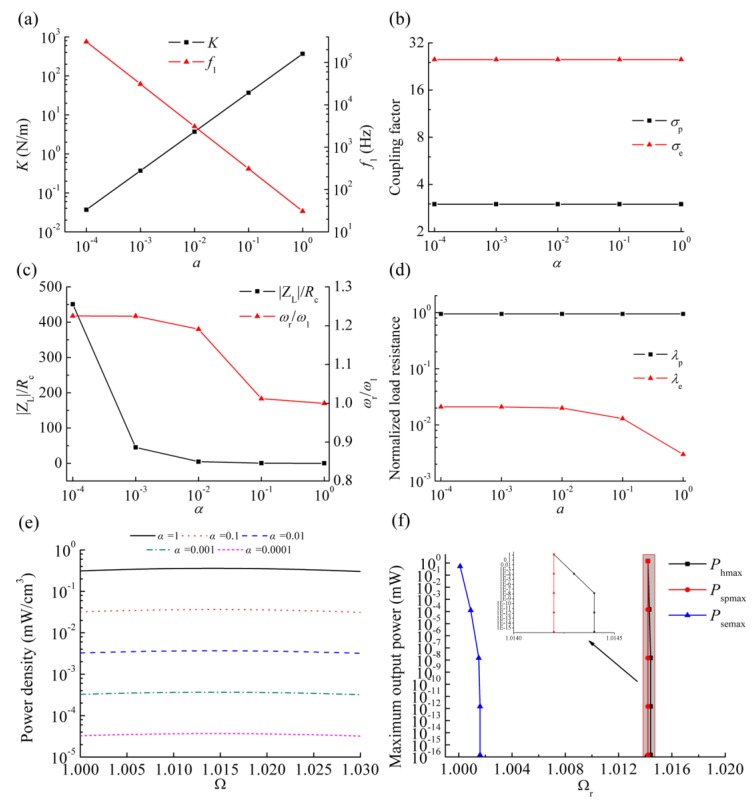
Performance of the HEH for different scaling factor *α*: (**a**) effective stiffness and undamped resonant frequency; (**b**) coupling factor ratio; (**c**) ratios of |Z_L_|/*R*_c_ and *ω*_r_/*ω*_1_; (**d**) normalized load resistances; (**e**) power density; (**f**) Maximum output power and resonant frequency ratios for HEH, stand-alone PEH and stand-alone EMEH.

To observe the size-scale effect on the performance, we hold all parameters constant except the geometric dimensions. Define *α* as the scaling factor. As a sample, all geometric dimensions of the HEH are scaled down by 100 times when *α* = 0.01. Five cases with scaling factors of 1, 0.1, 0.01, 0.001 and 0.0001 are calculated to explore the scaling effects. That is to say, the performance of the HEH is analyzed with the size range from the mm to the μm scale. Figure 9 displays the mechanical and electrical performance of the HEH for different scaling factors. Figure 9a shows a positive correlation between the effective stiffness and scaling factor. Therefore, the beam can deform more and more under the same transverse load as the size is scaled down. However, the undamped resonant frequency *f*_1_ of the piezoelectric beam increases with the size reduction. That is due to greater shrinking of the mass by *α*^3^. Piezoelectric and electromagnetic coupling factors have no size dependence under the assumption that the material parameters remain unchanged. As a resonant VEH, it is clear that only in the vicinity of the resonant frequency can the HEH have excellent generating performance. Therefore, the inductive reactance |Z_L_| at undamped resonant frequency and its effect on the resonant frequency *ω*_r_ are analyzed, as illustrated in Figure 9c. Because of the increment of undamped resonant frequency, the ratio of |Z_L_| to *R*_c_ gradually increases with the size scaling down. Although inductive reactance is enhanced, the ratio of *ω*_r_ to *ω*_1_ rises first, then levels off when *α* is less than 0.001. We can find the reason from Equation (22):

(22) $$\frac{\omega_{r}}{\omega_{1}}=\sqrt{1+\kappa_{e}^{2}\cdot \frac{Z_{L}^{2}}{Z_{L}^{2}+{\left(R_{2}+R_{c}\right)}^{2}}}$$

When *α* is less than 0.001, |Z_L_| is much larger than *R*_c_. Generally, *R*_2_ and *R*_c_ have the same order of magnitude. Consequently, this ratio gradually approaches $\sqrt{1+\kappa_{e}^{2}}$, which is not affected by the size scaling. The normalized load resistance *λ*_p_ keeps constant, while *λ*_e_ increases first, then levels off when *α* is less than 0.01.

To compare the output power with different size scale, we investigate the power density (PD) of the HEH for different scaling factors and excitation frequency ratios Ω. As shown in Figure 9e, PD is proportional to the scaling factor. Therefore, it is a challenging issue to improve the PD of MEMS-scale VEH. Figure 9f shows the maximum output power of the HEH (*P*_hmax_), stand-alone PEH (*P*_spmax_) and stand-alone EMEH (*P*_semax_) with matched load resistances and resonant frequency ratios Ω_r_ for different *α*. As the induction coil is open-circuit and only *R*_1_ is connected, the HEH is changed into a stand-alone PEH. When *R*_2_ is connected to the induction coil and the piezoelectric layers are directly connected without external load resistance (short-circuit condition), the stand-alone EMEH is developed. Firstly, there is almost the same trend for the resonant frequencies of the HEH and stand-alone EMEH, which increase with the size scaling down and then remain stable. The resonant frequency of the stand-alone PEH is not affected. All maximum output power drops dramatically as the size decreases. Note that the value of *P*_hmax_ is greater than that of *P*_spmax_ and *P*_semax_ for any *α*, based on the geometric parameters listed before. It demonstrates again that hybrid energy harvesting can enhance the output power of the VEH even at the MEMS scale.

## 4. Experiment

**Figure 10:**
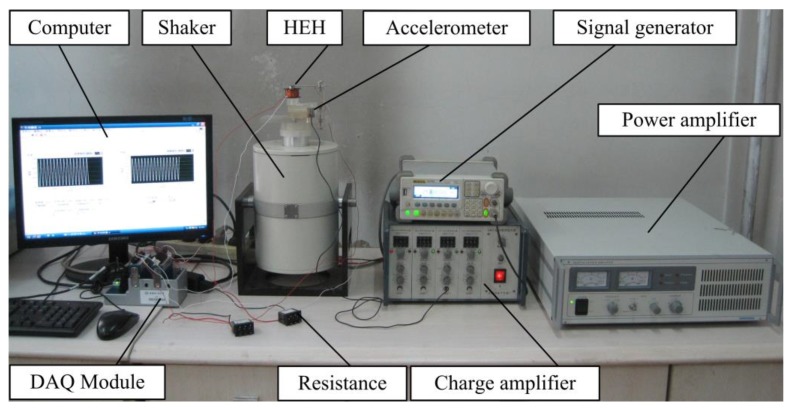
Experimental setup for the HEH.

In order to verify the established theoretical model, an experimental test system was assembled, as shown in Figure 10. A sinusoidal-wave excitation signal is generated by a signal generator (DG-1022, Rigol Technologies Inc., Beijing, China), amplified by a power amplifier (YE5874A, Sinocera Piezotronics Inc., Yangzhou, China) and used to control the vibration of an electromagnetic shaker (JZK-50, Sinocera Piezotronics Inc., Yangzhou, China). The vibration frequency and acceleration are acquired by an accelerometer (YD64-310, Qinhuangdao Xinheng Electronic Technology Co., Ltd., Qinhuangdao, China) and conditioned by a charge amplifier (CA-3, Qinhuangdao Xinheng Electronic Technology Co. Ltd., Qinhuangdao, China). The generated voltage and acceleration signal are input into a computer through the data acquisition module (NI 9229, National Instruments Co., Austin, TX, USA).

**Figure 11:**
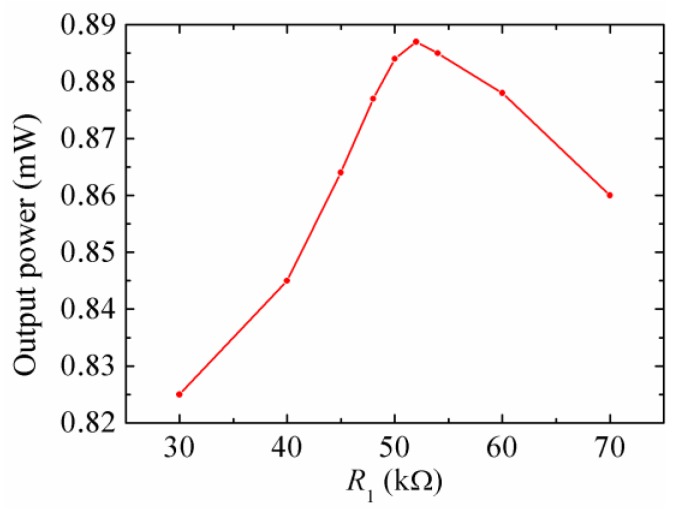
The output power of the stand-alone PEH for different *R*_1_ at the resonant frequency.

Based on the previous analysis, the output power of the HEH is optimal when the load resistances connected are matched. In order to compare the output power frequency responses of the stand-alone PEH, stand-alone EMEH and HEH, it is necessary to respectively match the load resistances. Figure 11 shows the output power of the stand-alone PEH for different *R*_1_ at the resonant frequency. The base excitation was harmonic, and acceleration was kept at 2 m/s^2^. Through the experimental test, the output power achieved the maximum 0.887 mW when the matched resistance *R*_1_ was 52 kΩ. When *R*_1_ was reduced to 1 kΩ, the piezoelectric layers were considered to be short-circuit connected, and the piezoelectric damping was negligible. Under this condition, the mechanical damping ratio of the piezoelectric beam was measured to be 0.0295 by using the logarithmic decrement method.

**Figure 12:**
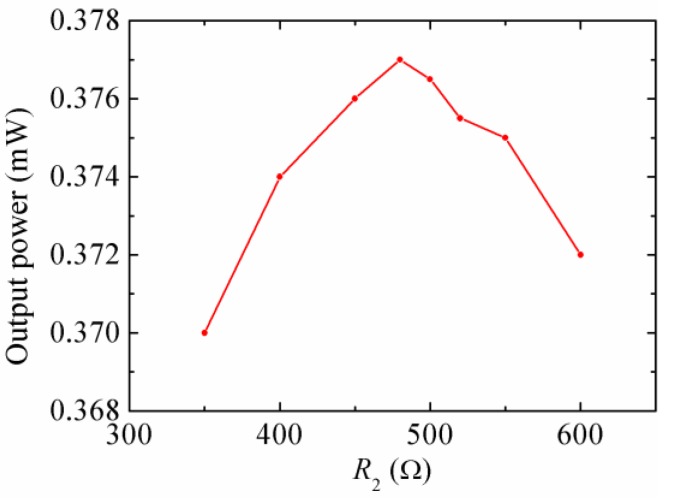
The output power of the stand-alone EMEH for different *R*_2_ at the resonant frequency.

Figure 12 represents the output power of the stand-alone EMEH for different *R*_2_ at the resonant frequency. The output power reaches the peak 0.377 mW when *R*_2_ was equal to 480 Ω. The measured internal resistance of the coil was 338 Ω. Letting *R*_2_ = 10 kΩ produced an approximately open-circuit condition for the coil. The mechanical damping ratio of the magnetic oscillator was measured to be 0.025. Compared with that of the piezoelectric oscillator, the change of mechanical damping ratio is mainly due to the secondary piezoelectric effect of the piezoelectric material, which induces more damping to the system.

**Figure 13:**
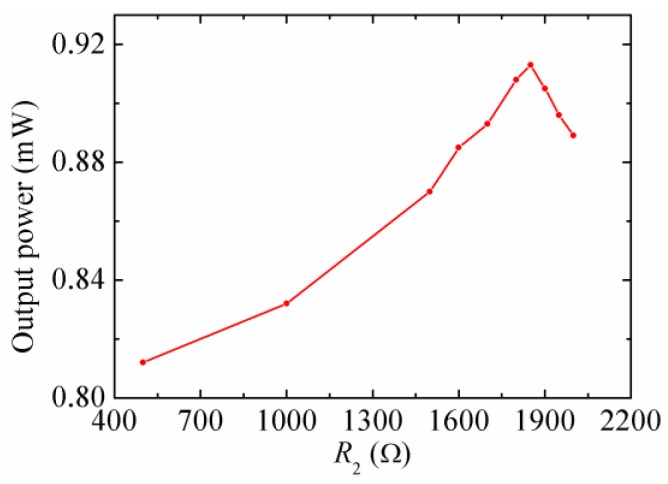
The output power of the HEH for different *R*_2_ at the resonant frequency.

Figure 13 illustrates the output power of the HEH for different *R*_2_ at the resonant frequency, when the piezoelectric layers were connected with the matched *R*_1_. The maximum power 0.913 mW occurs when *R*_2_ = 1850 Ω at the resonant frequency. It can be seen that the matched *R*_2_ is different from that of the stand-alone EMEH. This is because the resonant frequency of the beam increases when matched *R*_1_ is connected to the piezoelectric layers, which results in the change of the matched *R*_2_.

**Figure 14:**
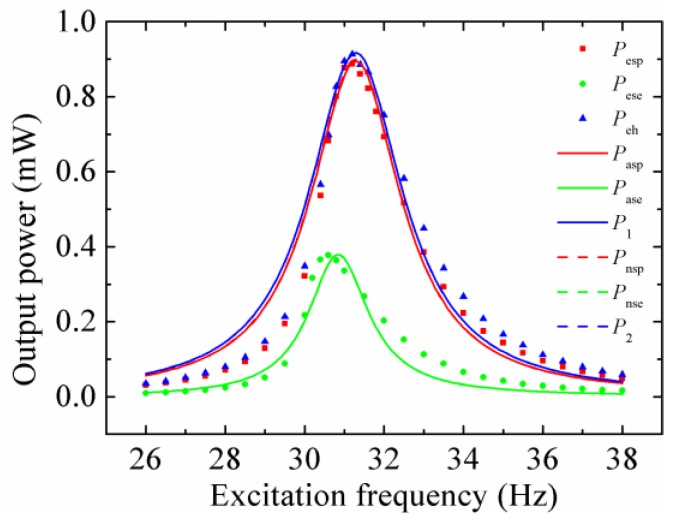
The optimal output power of the stand-alone PEH, stand-alone EMEH and HEH at different excitation frequencies.

At last, the optimal output power of the stand-alone PEH, stand-alone EMEH and HEH at different excitation frequencies was tested in turn, as shown in Figure 14. Note that the subscripts “esp”, “ese” and “eh” represent the experimental results of the stand-alone PEH, stand-alone EMEH, and HEH, respectively. “asp” and “ase” stand for the analytical results of the stand-alone PEH and stand-alone EMEH, respectively. “nsp” and “nse” mean the numerical results of the stand-alone PEH and stand-alone EMEH, respectively. The load resistances corresponding to the optimal output power were the matched ones. It can be seen that the numerical results nearly coincide with the analytical results. The experimental results also show good agreement with the theoretical values. It proves that the theoretical model is valid. The measured peak output power of the HEH (0.913 mW) is 2.93% and 142.18% higher than that of the stand-alone PEH (0.887 mW) and EMEH (0.377 mW), respectively. Moreover, the operation frequency bandwidth of the HEH is the widest, which (about 2.7 Hz) increases up to 108%- and 122.7%-times that of stand-alone PEH (about 2.5 Hz) and EMEH (about 2.2 Hz), respectively. Note that *σ*_p_ and *σ*_e_ of the HEH prototype are 2.03 and 17.44, respectively. Consequently, the superiority of the hybrid energy harvesting mechanism is in accordance with the previous theoretical analysis. The measured resonant frequency of the HEH is 31.2 Hz, which is the same as that of the stand-alone PEH, but higher than that of the stand-alone EMEH (30.6 Hz). It indicates that coupling electromagnetic energy harvesting has little effect on the system resonant frequency. In addition, the tested resonant frequencies are lower than the theoretical values, which is mainly caused by the softened spring effect of the resonant structure [32].

## 5. Conclusions

In order to evaluate the generating performance of the piezoelectric-electromagnetic hybrid energy harvester, this paper developed an approximate distributed-parameter theoretical model of the HEH based on the energy method and Euler–Bernoulli beam theory. The analytical solutions were compared with the numerical solutions and used to observe the influences of mechanical and electric parameters on the generating characteristics and dynamic responses of the HEH. A meso-scale HEH prototype was fabricated and tested. The experimental results verified the theoretical model and analysis. The following conclusions were obtained:

The difference between numerical and analytical solutions gradually increases with the increment of excitation acceleration. The load resistance connected to piezoelectric layers has a significant effect on tuning the resonant frequency of the HEH, while the effect of that connected to the coil can be neglected. Therefore, the load resistance of the piezoelectric energy harvesting subsystem should be preferentially determined during the impedance matching of the experiment. The matched load resistances for the optimal output power and energy conversion efficiency are not consistent. Extracting electrical energy can suppress the vibration of beam. However, the optimal output power and energy conversion efficiency do not imply an optimal vibration suppression effect. Regardless of the piezoelectric coupling strength, coupled electromagnetic and piezoelectric energy harvesting results in widening operating frequency bandwidth and improving the energy conversion efficiency. However, the HEH generates more power than the single-mechanism energy harvester only when the piezoelectric coupling effect is weak or medium. The piezoelectric coupling factor of the HEH prototype is 2.03, which denotes the medium coupling. Its maximum output power (0.913 mW) with matched load resistances is 2.93% and 142.18% higher than that of the stand-alone PEH (0.887 mW) and EMEH (0.377 mW), respectively. The operation frequency bandwidth of the HEH is 108%- and 122.7%-times of that of stand-alone PEH and EMEH, respectively. The superiority of the HEH on the output power to the stand-alone PEH is also affected by the mechanical damping ratio. The influence of the inductive coil on the system resonant frequency can be neglected when the frequency of the vibration source is less than 200 Hz. For the optimal output power, the coil parameters of the HEH should be optimized according to the electromagnetic coupling factor. When the size is scaled down to the micro scale, some mechanical and electrical performance is affected. However, it indicates that hybrid energy harvesting can enhance the output power of the VEH even at the MEMS scale.

The numerical model can be used for the optimization of the HEH to harvest maximum power from a given excitation source. However, the accuracy of the analytical model can be guaranteed at the low-level excitation acceleration. Furthermore, it has a better computational efficiency. The superiority of the HEH relative to the stand-alone PEH or EMEH depends on mechanical and electric parameters. In future work, an integrated system including HEH and interface circuit will be designed, analyzed and optimized.

## Figures and Tables

**Table 1:** Geometric and material parameters of the HEH.

Parameter	Value
Substrate length × width × thickness (mm^3^) *L* × *b* × *h*_s_	62 × 20 × 0.26
Substrate density (kg/m^3^) *ρ*_s_	8920
Substrate Young’s modulus (GPa) *c*_s_	90
PZT length × width × thickness (mm^3^) *L* × *b* × *h*_p_	50 × 20 × 0.2
PZT density (kg/m^3^) *ρ*_p_	7386
PZT elastic stiffness (GPa) $c_{11}^{E}$	59.77
Piezoelectric stress constant (C/m^2^) *e*_31_	−13.74
Dielectric permittivity (nF/m) $\varepsilon_{33}^{S}$	38.62
Magnet radius × height (mm^2^) *R* × *h*_m_	6 × 10
Residual magnetic flux density (T) *B_r_*	1.25
Magnet density (kg/m^3^) *ρ*_m_	7800
Coil turns *N*	2050
Coil inner radius × outer radius × height (mm^3^) *R*_i_ × *R*_o_ × *h*_c_	11 × 13.15 × 17
Wire diameter (mm) *D*_w_	0.123
Wire resistance per unit length (Ω/m) *ρ*_c_	2.16

**Table 2:** Comparison of peak power from numerical and analytical solutions.

Acceleration (m/s^2^)	Numerical Results				Analytical Results			
	Ω_r_	Peak Power (mW)			Ω_r_	Peak Power (mW)		
		*P*_1_	*P*_np_	*P*_ne_		*P*_2_	*P*_ap_	*P*_ae_
2	1.015	1.288	1.081	0.207	1.015	1.287	1.080	0.207
10	1.015	32.969	27.635	5.334	1.015	32.175	27.002	5.173
20	1.015	139.291	116.588	22.703	1.015	128.702	108.008	20.694
40	1.015	600.311	502.117	98.194	1.015	514.806	432.031	82.775
